# Factors Associated with Intimate Partner Violence Perpetration Among Migrant Men: A Systematic Review

**DOI:** 10.1177/15248380231178758

**Published:** 2023-06-10

**Authors:** Matin Ayubi, Lata Satyen

**Affiliations:** 1 Deakin University, Burwood, VIC, Australia

**Keywords:** batterers, domestic violence, cultural contexts, domestic violence and cultural contexts, domestic violence, cultural contexts, intervention/treatment, domestic violence, mental health and violence, violence exposure

## Abstract

Intimate partner violence (IPV) is the most widespread form of violence against women and the most common perpetrators are male partners. Immigration can involve stressors and barriers that are linked to male IPV perpetration. The objective of this systematic review was to identify the factors associated with IPV perpetration among migrant men. Four electronic databases, MEDLINE Complete, Embase, PsycInfo, and SocINDEX with full text, were searched up to August 2021. Studies were selected that examined factors associated with IPV perpetration among first-generation migrants who identified as men/males and were aged 18 years or older. In all, 18 articles met the eligibility criteria for the review, representing a total of 12,321 male participants, including 4,389 migrant men. A wide range of factors associated with IPV perpetration were found at the individual, relationship, community, and societal levels. Unique risk factors for migrant men’s IPV perpetration were exposure to political violence, deportation experiences, and minimal legal sanctions for perpetration in some countries of origin. Societal factors explored among Latino immigrants were traditional gender roles such as *machismo* and norms of violence. All identified factors should be considered in the cultural contexts of the relevant samples and should not be generalized to all migrant men. The findings of modifiable and culture-specific factors have important implications for strategies aimed at reducing IPV perpetration. Future research should explore factors associated with IPV perpetration within specific cultures rather than across broad cultural groupings.

Intimate partner violence (IPV, also referred to as domestic violence or dating violence) involves acts of physical, sexual, psychological, and economic/financial abuse within an intimate relationship (World Health Organization [WHO], 2021). IPV is the most widespread form of violence against women; global estimates indicate that over a quarter (27%) of women aged 15 to 49 years have been physically and/or sexually abused by a current or former intimate partner (WHO, 2021). IPV is associated with harm and negative outcomes for women, including injury, chronic pain, depression, social isolation, unemployment, and homelessness (Satyen, Supol, et al., 2021; Stubbs & Szoeke, 2022).

Male intimate partners are the most common perpetrators of violence against women (WHO, 2021). In Asia and the Pacific, rates of physical and/or sexual violence perpetration by male intimate partners ranged from 25.4% to 80.0% (Fulu et al., 2013). Women’s experiences of IPV victimization vary across world regions; lifetime IPV rates were highest in Africa (33%), followed by Oceania (30%), Asia (27%), the Americas (25%), and lowest in Europe (20%; WHO, 2021). Given the high rates of male IPV perpetration and varying IPV rates worldwide, it is important to understand the range of factors that influence such violence.

## Immigration and IPV

The impact of immigration on IPV requires further exploration, as emphasized by estimates that 272 million international migrants, refugees, and asylum seekers account for 3.5% of the world’s population (McAuliffe & Khadria, 2020). Research suggests that migrant women experience high rates of IPV victimization, with prevalence estimates of victimization ranging from 17% to 70.5% (Gonçalves & Matos, 2016). The literature consists of mixed findings on whether migrants are at increased risk of IPV compared to non-migrants. Studies conducted in the United States and Spain found that IPV was more prevalent among immigrants (Sanz-Barbero et al., 2019; Vaughn et al., 2015), whereas an Australian study showed that rates of IPV victimization were similar for migrant and non-migrant women (Satyen, Toumbourou, et al., 2021). These contrasting results indicate that IPV experiences of migrants vary depending on the host country. Comparisons between cultural groups within the same host country demonstrate further complexity. For example, in the United States, Latin American immigrants (8.99%) had the highest rates of IPV perpetration and were more likely than U.S.-born Americans (7.34%) to perpetrate IPV, whereas immigrants from Asia (5.72%), Africa (3.51%), and Europe (3.33%) were less likely to perpetrate IPV compared to U.S.-born Americans (Vaughn et al., 2015). Taken together, the findings support the notion that immigrants are a heterogeneous group and IPV experiences will vary depending on the host country and cultural group.

While the global immigrant population is heterogeneous, that is, originating and resettling in different countries and having many different reasons for migration, there are shared characteristics, stressors, and barriers which may increase their vulnerability to IPV (Gonçalves & Matos, 2016). The migration and resettlement process involves multiple stressors and barriers such as the dispersion of households, changes to family dynamics, loss of social networks and social support, unemployment, discrimination in the job market, financial and status change, language and communication problems, and racism and stereotyping (Dow, 2011; S. S. Y. Li et al., 2016; Porter & Haslam, 2005). In Farrington’s (1986) stress theory, IPV is viewed as a coping behavior that occurs when the demands posed by stress exceed the individual’s response capabilities. Given the broad range of stressors experienced by migrant men, and the possibility that these stressors are being experienced for the first time, migrant men may be at increased risk of having their response capabilities overwhelmed and consequently perpetrating IPV.

Correspondingly, immigration can increase the risk of IPV victimization for migrant women. Factors such as uncertain legal status, limited host-language skills, lack of social support, and poverty can increase women’s vulnerability to IPV victimization (Gonçalves & Matos, 2016; Vaughn et al., 2015). For migrant women who experience IPV victimization, there are many barriers to disclosure and help-seeking; for example, migrant women may be inhibited from reporting IPV due to language and financial barriers, experiences of racial discrimination, stigma toward help-seeking, and fear of deportation and losing their children (Gonçalves & Matos, 2016; Satyen, Supol, et al., 2021). Barriers to help-seeking for migrant women include lack of knowledge about services, lack of awareness of their rights, accessibility issues, and economic dependence on their partners (Gonçalves & Matos, 2016; Satyen, Supol, et al., 2021; Vaughn et al., 2015).

## Male IPV Perpetration in the General Population

Research shows that male IPV perpetration in the general population is a complex interplay of individual, relationship, and contextual factors. The strongest risk factors for male IPV perpetration have been related to other acts of violence, including past IPV perpetration, past IPV victimization, and causing previous injury to one’s partner (C. M. Spencer et al., 2022). Men’s mental ill health has been associated IPV perpetration, particularly depression, anxiety, post-traumatic stress disorder, personality disorders, substance use, anger, and jealousy (Clare et al., 2021; C. Spencer et al., 2019; C. M. Spencer et al., 2022). Experiencing childhood trauma in the form of childhood family violence (CFV), including witnessing parental IPV and being abused as a child, has a significant impact on adult IPV perpetration (Capaldi et al., 2012; Kimber et al., 2018; S. Li et al., 2020). Social learning theory suggests that children who are exposed to CFV learn the aggressive and violent behaviors and the consequences of such behaviors, both positive (e.g., compliance from the victim) and negative (e.g., divorce, further conflict; Bandura, 1973; Voith et al., 2020). Other factors associated with IPV perpetration relate to the intimate relationship, including men’s controlling behaviors, gender-inequitable attitudes, and demand/withdraw relationship patterns, and social markers such as low education levels, poverty, prior arrest, and access to firearms (Clare et al., 2021; Fulu et al., 2013; C. M. Spencer et al., 2022). Collectively, these risk factors demonstrate the broad scope and complexity of factors underlying IPV perpetration.

## Male IPV Perpetration Among Migrants

There is a dearth of research on IPV perpetration among migrant men, but several factors have been identified. Migrant men may be more predisposed to perpetrating IPV due to cultural factors from the country of origin which condone IPV, such as traditional gender roles, patriarchal values and inequalities, and positive attitudes toward violence (Gonçalves & Matos, 2016; Satyen, Supol, et al., 2021; Vaughn et al., 2015). Post-migration factors in the host country which have been associated with IPV perpetration among migrant men include acculturation stress, integration levels, and work-related stress (Caetano et al., 2007; Jasinski et al., 1997; Vaughn et al., 2015). Migrant IPV perpetrators in the United States were significantly more likely than non-perpetrators to meet criteria for mood, anxiety, personality, and substance use disorders, and it has been suggested that cultural norms and acculturation stress may amplify the effects of these disorders for migrant men (Vaughn et al., 2015). Among asylum seekers and refugees specifically, male IPV perpetration was associated with younger age, lower education levels, refugee status, direct exposure to political violence, mental health problems, tolerant attitudes toward violence, and relationship factors, such as the husband’s controlling behaviors and marital conflict (El-Moslemany et al., 2020).

### The Role of Trauma

Given the evidence for the impacts of exposure to violence and mental health problems on IPV perpetration among migrants, it is essential to consider the role of trauma. Trauma is defined as experiencing an extreme or disturbing event that provokes intense and disruptive feelings, resulting in a long-lasting negative impact on an individual’s attitudes, behavior, and other aspects of functioning (American Psychiatric Association, 2022). Migrants can be exposed to multiple traumas throughout their migration journey, such as conflict, physical and sexual violence, persecution, war, and economic and political instability (McAuliffe & Khadria, 2020; Theisen-Womersley, 2021). High rates of exposure to political violence have been demonstrated among migrant men (Rousseau & Drapeau, 2004), and political- and war-related violence has been associated with an increased likelihood of IPV perpetration (Sousa et al., 2018; Vinck & Pham, 2013). Among refugee couples, the trauma of war and forced migration have been linked to increased levels of marital conflict (Doha International Family Institute, 2018).

### The Role of Patriarchy

A key theoretical concept in feminist literature on violence against women is patriarchy, which refers to systems of male domination and female subordination (Hunnicutt, 2009). Male IPV perpetration is viewed as a product of social-structural conditions that provide men with more power than women, rather than the perpetrator’s attributes and motivations (Hunnicutt, 2009). Limited research exists on cross-cultural comparisons of patriarchal values and their association with IPV perpetration. Where there is such research, it shows that countries and cultures vary in patriarchal values (Grossman & Lundy, 2007), and that male IPV is more likely to be perpetrated and socially accepted in societies with stronger patriarchal orientations (Archer, 2006; Fulu et al., 2013; Pichon et al., 2020). For example, a cross-cultural comparison demonstrated that Asian men were significantly more likely than European men to endorse patriarchal values and perpetrate severe physical IPV (Ozaki & Otis, 2017). Similarly, research on patriarchal cultural norms in China, Japan, and South Korea showed that a belief in male dominance and violence approval significantly predicted male IPV perpetration (Ozaki & Otis, 2017). In Ghana, male IPV perpetration was related to beliefs about men having the right of decision-making, rigid and distinct gender roles, ownership over their partners, and IPV being legitimate discipline (Sikweyiya et al., 2020). There are also significant impacts on men’s IPV perpetration in cultures (e.g., Bangladesh and Cambodia) where inequitable attitudes to gender are more prevalent (Fulu et al., 2013).

Migration can alter patriarchal values of migrant men through changes in relationship dynamics of power and dominance. For example, traditional gender roles may be affected when migrant women enter the workforce and earn a wage, and migrant men have a weaker role as the main or sole financial provider (Min, 2001; Raj & Silverman, 2002). These changes in gender roles post-migration can threaten male dominance and the patriarchal values of their cultures of origin, leading to increased efforts to control their partners through IPV perpetration (Min, 2001; Raj & Silverman, 2002). Therefore, migration may increase the risk of IPV perpetration for men who come from more patriarchal cultures.

## Objective and Framework of the Review

Given the large numbers of migrants worldwide and the range of stressors associated with immigration, it is important to better understand IPV perpetrated by migrant men to aid prevention and intervention efforts. To date, there are no published systematic reviews on IPV perpetration and associated factors among the broad group of migrant men. Recently, El-Moslemany et al. (2020) conducted a systematic review on factors associated IPV perpetration and victimization in asylum seeking and refugee populations. The present systematic review expands on previous research by including all migrants who have crossed an international border and by focusing exclusively on male IPV perpetration. Thus, the objective of this systematic review was to identify the factors associated with IPV perpetration among migrant men.

The factors associated with IPV are conceptualized within an ecological model, which was first developed by Bronfenbrenner (1992) to explain child development, and later adapted to understand violence (Krug et al., 2002). The ecological model contends that violent behavior cannot be explained by any single factor and is instead the product of multiple levels of influence from individual and contextual factors (Krug et al., 2002). The ecological model for understanding violence is comprised of four interconnected levels: individual, relationship, community, and societal (Krug et al., 2002). Individual factors are specific characteristics of the individual which may increase their likelihood of being a perpetrator, such as biological and sociodemographic factors. Relationship factors explore the impact of proximal social relationships, such as relations with intimate partners, peers, and family members. Community factors examine contexts in which social relationships are embedded, such as workplaces and neighborhoods, and characteristics of communities that increase the risk of violence, including poverty and lack of structural supports. Societal factors include attitudes, cultural norms, and policies which influence violent behavior, both in the perpetrator’s country of origin and in the host country.

## Method

### Eligibility Criteria

Inclusion criteria required participants to identify as a man/male, to be aged 18 years or older, and to be a first-generation migrant, refugee, or asylum-seeker who had crossed an international border. For the purpose of this review, the terms *migrant* and *immigrant* were used synonymously to refer to individuals who had left their country of origin and crossed an international border to live in a new country. Studies were excluded if participants were not separated by sex or gender, were under the age of 18 years, were internal migrants, internally displaced people, or second-generation migrants and beyond. Studies were required to include IPV perpetration as a variable and to explore its association with another variable. Studies needed to include a self-report measure of IPV perpetration or recruit participants convicted of perpetrating IPV. Studies were excluded if they included partner reports of IPV in the analyses and did not separate results by gender. Studies could examine IPV within a heterosexual or same-sex relationship. The presence of a comparison group was not required for inclusion.

Experimental, observational (including cohort, cross-sectional, and case–control studies), and qualitative studies were included. Reports, conference abstracts, discussion papers, editorials, letters, and gray literature were excluded. Only peer-reviewed articles published in English were included. Limitations were not placed on publication year, and no time limits were placed on when exposures or outcomes were assessed.

### Search Strategy, Selection Process, and Data Collection

Four electronic databases were searched for eligible studies from inception to August 2021: MEDLINE Complete, Embase, PsycInfo, and SocINDEX with full text. Reference lists of included articles and previously published systematic reviews were hand-searched to identify additional relevant articles. A comprehensive searched strategy covered the concepts of men, migrants, IPV, and perpetration. Factors associated with IPV were not included as a concept because this would have limited the results. The included concepts were searched using a combination of free-text terms and controlled vocabulary specific to each database (see Supplemental Appendices A–F).

Citations identified from the database searches were imported in EndNote and duplicates were removed. The remaining records were imported in Covidence for screening. One reviewer (MA) screened titles and abstracts of all articles retrieved, and then two reviewers (MA and LS) independently screened full-text articles for inclusion. In case of disagreement, consensus was reached on inclusion or exclusion by discussion. This process was repeated with citations identified from a hand-search of the reference lists of included articles and previously published systematic reviews. A data extraction form was developed in Microsoft Excel by both reviewers (MA and LS). Data were collected by one reviewer (MA) and independently checked for accuracy by a second reviewer (LS). Any discrepancies were resolved through discussion.

### Study Risk of Bias Assessment

Given the range of study designs in the included studies, we assessed risk of bias using three tools: The National Heart, Lung, and Blood Institute’s (NHLBI, 2021) Quality Assessment Tool for Observational Cohort and Cross-Sectional Studies, NHLBI’s (2021) Quality Assessment of Case-Control Studies, and the Critical Appraisal Skills Programme’s (CASP, 2018) Qualitative Studies Checklist. The NHLBI (2021) tools evaluate the internal validity of a study by testing for potential flaws in study methods or implementation and most criteria refer to exposure and outcome variables. The CASP (2018) Qualitative Studies Checklist is a 10-item tool that considers the validity of the results, the content of the results, and the value of the research. The quality assessment was conducted by one reviewer (MA) and independently checked for accuracy by a second reviewer (LS), with any discrepancies resolved by consensus. For all tools, assessment was specific to evidence relevant to this review, and not the overall quality of the studies. The importance of each criterion for the review subject was considered in determining overall ratings of poor, fair, or good. Higher number of satisfied criteria generally correspond to higher quality ratings and only relevant criteria were considered when determining the quality ratings.

## Results

### Study Selection

**Figure 1.**
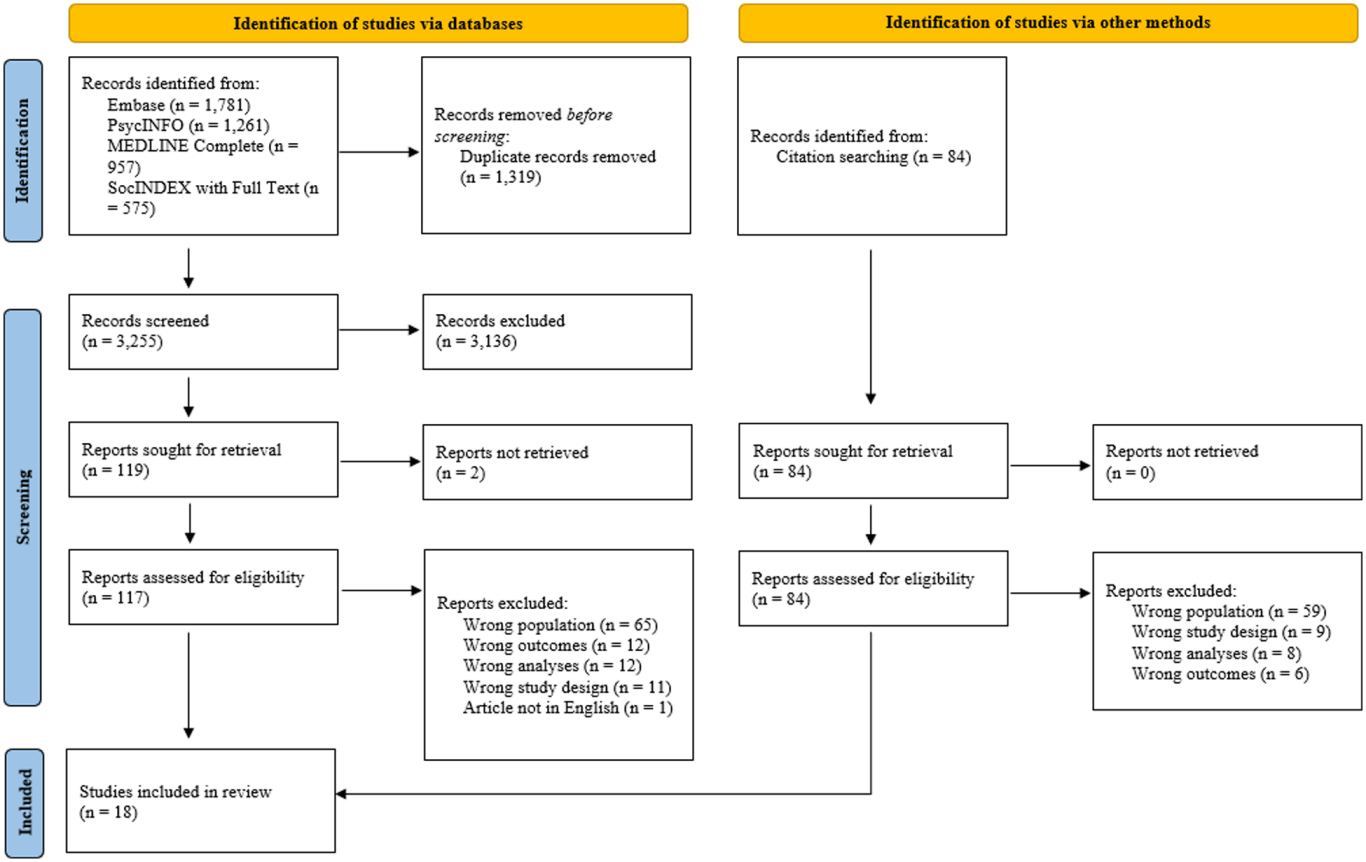
Preferred Reporting Items for Systematic Reviews and Meta-Analyses flow diagram for study selection. *Source*. Adapted from Page et al. (2021).

The database searches were conducted in August 2021 and yielded 4,574 records. After duplicates were removed, 3,255 records were screened, from which 117 full-text documents were retrieved and reviewed. From this process, 16 papers were included in the present review (Baker et al., 2001; Edelstein, 2018; Fernández-Montalvo et al., 2022; Gilbert et al., 2019; Gupta et al., 2009, 2010; Jin & Keat, 2010; Jin et al., 2007, 2008; J. Y. Kim & Sung, 2000; I. J. Kim & Zane, 2004; Maldonado et al., 2020; Montalvo-Liendo et al., 2018; Rothman et al., 2007; Saez-Betacourt et al., 2008; Welland & Ribner, 2010). Reference lists of included articles and previously published systematic reviews were hand-searched and the full texts of 84 potentially eligible records were reviewed. Two extra articles that fulfilled the criteria were included (Grzywacz et al., 2009; Nam et al., 2020), resulting in a total of 18 studies in the review. A flow diagram for the study selection process is presented in Figure 1 in accordance with the updated Preferred Reporting Items for Systematic Reviews and Meta-Analyses (PRISMA) 2020 statement (Page et al., 2021).

Among the full-text articles assessed for eligibility from database searching and citation searching, 184 studies were excluded from the review. Reasons for exclusion were wrong study population (e.g., participants were not adult male migrant IPV perpetrators), wrong outcomes (e.g., IPV was not included as a variable in the study), wrong analyses (e.g., lack of separate analyses for migrant men only or perpetrators only), wrong study design (e.g., not original research), and articles not being published in English.

### Study Characteristics

The methodological characteristics of the included studies and the characteristics of the relevant migrant and comparison groups are summarized in Table 1. Studies are cited in text only when information is not included in tables or appendices. Publication dates spanned 22 years, from 2000 to 2022. The majority of studies (13; 72%) had a cross-sectional design, four studies (22%) were qualitative, and one study (6%) was case-controlled. For their migrant samples, 17 studies (94%) recruited immigrants and one study (6%) recruited refugees. Sample sizes of the studies ranged from eight to 3,460 participants, and the studies included a total of 12,321 male participants, of which 4,389 were migrant men.

**Table 1.** Methodological Characteristics of Included Studies.

Author (Year)	Location	Research Design	*N*	Males (%)	Characteristics of Migrants Relevant to the Review			Control/Comparison Group Characteristics, Percentage of Total Sample
					Age, *M* (*SD*) or Range	Type of Migrant, Percentage of Total Sample	Country/Region of Origin or Race/Ethnicity, Percentage of Migrant Group	
Baker et al. (2001)	USA	Cross-sectional	86	50	29.76 (4.29)	Immigrants 60.5%	Mexico 85%	Community sample of Latino immigrants 39.5%
Edelstein (2018)	Israel	Case–control	194	100	N/R	Immigrants 16%	Ethiopia	Israeli femicide perpetrators 47%
Fernández-Montalvo et al. (2022)	Spain	Cross-sectional	1,421	100	37.49 (10.68)	Immigrants 53.1%	N/R	Perpetrators with Spanish nationality 46.9%
Gilbert et al. (2019)	Kazakhstan	Cross-sectional	1,342	100	28.1	Immigrants 37%	Tajik 39.5%, Kazakh 31.3%, Kyrgyz 15.2%, Karakalpak 11.0%, Uzbek 2.0%, Uighur 0.2%, Other or multiethnic 1.0%^a^	Community sample of non-migrants 42% and internal migrants 21%
Grzywacz et al. (2009)	USA	Qualitative	20	50	20–52^b^	Immigrants 100%	Mexico	N/A
Gupta et al. (2009)	USA	Cross-sectional	379	100	25.9	Immigrants 100%	Caribbean 43.1%, Africa 40.2%, South America, Central America, and Mexico 7.2%, Other 9.6%	N/A
Gupta et al. (2010)	USA	Cross-sectional	1,668	100	25.8	Immigrants 26.5%	Black (non-Hispanic) 48.4%, Hispanic 41.9%, Asian and Native Hawaiian/Pacific Islander 2.6%, Native American/Alaska Native 2.1%, White (non-Hispanic) 1.9%, Other 3.2%^c^	Community sample of non-migrants 73.4%
Jin et al. (2007)	USA	Cross-sectional	126	100	40.74 (8.70)	Immigrants 51%	China	Community sample of Chinese immigrants 49%
Jin et al. (2008) ^d^	USA	Cross-sectional						
Jin and Keat (2010) ^d^	USA	Cross-sectional						
J. Y. Kim and Sung (2000)	USA	Cross-sectional	256	100	45	Immigrants 100%	Korea	N/A
I. J. Kim and Zane (2004)	USA	Cross-sectional	102	100	40.7 (9.8)	Immigrants 51%	Korea	European-American perpetrators 49%
Maldonado et al. (2020)	USA	Cross-sectional	2,287	100	41.00 (0.79)	Immigrants 52%	Latino	Community sample of non-migrant Latinos 48%
Montalvo-Liendo et al. (2018)	USA	Qualitative	8	100	18–40	Immigrants 25%	Mexico	Mexican American perpetrators 75%
Nam et al. (2020)	South Korea	Cross-sectional	998	100	19+	Refugees 10%	North Korea	Community sample of South Koreans 90%
Rothman et al. (2007)	USA	Cross-sectional	3,460	100	34.1	Immigrants 14%	Hispanic 36%, Black (non-Hispanic) 25%, White (non-Hispanic) 20%, Asian 9%, Other 9%	Non-migrant perpetrators 86%
Saez-Betacourt et al. (2008)	USA	Qualitative	15	100	35 (7.4)	Immigrants 100%	Mexico 87%, Guatemala 13%	N/A
Welland and Ribner (2010)	USA	Qualitative	12	100	N/R	Immigrants 100%	Mexico	N/A

*Note*. N/A = not applicable; N/R = not reported.

a Data extracted from El-Bassel et al. (2016).

b Total sample including women.

c Calculated using data from Gupta et al (2010).

d Study used the same sample as Jin et al. (2007).

The characteristics of the exposure and outcome measures used in the studies and outcome data are summarized in Supplemental Appendix G. In total, 11 studies (61%) measured IPV perpetration using a version of the Conflict Tactics Scales (CTS; Straus et al., 1996). Among these, two reported on physical IPV only; three on physical and sexual IPV; four on physical and psychological IPV; and two on physical, psychological, and sexual IPV. In addition to administering the CTS, two studies (11%) used items from the Sexual Experiences Survey (Koss et al., 1987) to assess sexual violence, and one study (6%) used the Psychological Maltreatment of Women Inventory (Tolman, 1989) to assess psychological violence. One study (6%) measured IPV perpetration using the General Structured Interview of Batterer Men (Echeburúa & Fernández-Montalvo, 1998). Six studies (33%) did not administer a measure of IPV. Among these studies, three recruited participants from perpetration intervention programs (Rothman et al., 2007; Saez-Betacourt et al., 2008; Welland and Ribner, 2010), and thus IPV perpetration was assumed. For the remaining three studies, Montalvo-Liendo et al. (2018) recruited participants who personally disclosed a history of abuse against their intimate partner, Grzywacz et al. (2009) selected individuals who personally disclosed IPV perpetration or were identified as IPV perpetrators by the general community, and Edelstein (2018) examined court cases in which men were convicted of intimate partner homicide (IPH).

Rates of IPV perpetration among immigrant men varied across studies. Ten studies recruited participants that were court-referred for IPV perpetration, and thus all these immigrant men had perpetrated lifetime IPV. One such study showed that among a group of Chinese IPV perpetrators, 70% had perpetrated past-year IPV (Jin et al., 2007). Comparatively, rates of past-year IPV perpetration in community samples across four studies ranged in ascending order from: 5% of Latino immigrants (Maldonado et al., 2020), 6.3% of Korean immigrants (severe violence; J. Y. Kim & Sung, 2000), 17.9% of a diverse group of immigrants (Gupta et al., 2009), 18.0% of Korean immigrants (any violence; J. Y. Kim & Sung, 2000), and 57.1% of North Korean refugees (Nam et al., 2020). Comparisons by immigrant status showed that 16.6% of recent immigrants (5 years or less in the United States) had committed past-year IPV compared to 23.9% of non-recent immigrants (6 years or more in the United States; Gupta et al., 2010). In a community sample of immigrant market workers in Kazakhstan, 5.8% had perpetrated IPV in the past 6 months, and 9.8% had perpetrated IPV over their lifetime (Gilbert et al., 2019).

In all, 13 studies (72%) collected primary data (Baker et al., 2001; Fernández-Montalvo et al., 2022; Grzywacz et al., 2009; Gupta et al., 2009; Gupta et al., 2010; J. Y. Kim & Sung, 2000; I. J. Kim & Zane, 2004; Montalvo-Liendo et al., 2018; Saez-Betacourt et al., 2008; Welland & Ribner, 2010), including three studies which used the same sample (Jin & Keat, 2010; Jin et al., 2007, 2008). Five (28%) conducted analyses on secondary data: Gilbert et al. (2019) analyzed data from the Silk Road Health Project (El-Bassel et al., 2016) from 2009 to 2012; Maldonado et al. (2020) used cross-sectional data from Wave 2 (2004–2005) of the National Epidemiologic Survey on Alcohol and Related Conditions; Nam et al. (2020) utilized data from the 2010 Nationwide Survey on Domestic Violence in South Korea; Edelstein (2018) sampled all court decisions on IPH in Israel from 1990 to 2010; and Rothman et al. (2007) used data from 26 perpetrators intervention programs certified by the Massachusetts Department of Public Health from 2002 to 2004.

### Methodological Quality of Studies

The quality assessment of the 13 cross-sectional studies is presented in Supplemental Appendix H. Overall, one study was deemed to be of good quality and 12 to be of fair quality, as assessed with the NHLBI’s (2021) Quality Assessment Tool for Observational Cohort and Cross-Sectional Studies. The main strengths common in all cross-sectional studies (13; 100%) included: clearly stating the research question, specifying and defining the study population, recruiting participants from the same population and uniformly applying eligibility criteria, and using exposure and outcome measures that were clear and consistent. Quality assessment for the case–control study (Edelstein, 2018) was conducted using NHLBI’s (2021) Quality Assessment of Case–Control Studies. This study was of good quality, fulfilling 9 out of 12 criteria. The qualitative studies were assessed for their quality with the CASP (2018) Qualitative Studies Checklist. Three out of four studies (Grzywacz et al., 2009; Montalvo-Liendo et al., 2018; Saez-Betacourt et al., 2008) fulfilled all 10 criteria, indicating good methodological quality. One study fulfilled 7 out of 10 criteria (Welland & Ribner, 2010), indicating fair methodological quality.

### Results of Individual Studies and Syntheses

The key results from the 14 quantitative studies, including relevant statistics and *p* values, are presented in Table 2. The main themes from the four qualitative studies are displayed in Table 3. The factors associated with IPV are presented on four interconnected levels: individual, relationship, community, and societal.

**Table 2.** Significant Results of Included Quantitative Studies of Factors Associated with IPV Perpetration Among Migrant Men.

Authors (Year)	Country/Region of Origin	Host Country	Factor	Level	Results
Baker et al. (2001)	Mexico 85%	USA	Parenting competence	Relationship	Physical and sexual IPV: *r* = −.44, *p* < .01 Psychological IPV: *r* = −.52, *p* < .01
Edelstein (2018)	Ethiopia	Israel	Jealousy	Relationship	*p* < .05
Fernández-Montalvo et al. (2022)	N/R	Spain	CFV	Relationship	*OR* = 1.40; 95% CI [1.09, 1.80], *p* = .009
Gilbert et al. (2019)	N/R	Kazakhstan	Poor living conditions	Individual	aOR = 3.07; 95% CI [1.17, 8.11], *p* = .023
			Food insecurity	Individual	aOR = 4.37; 95% CI [1.72, 11.07], *p* = .002
			Exposure to political violence	Community	aOR = 15.08; 95% CI [3.39, 67.07], *p* < .001
			Deportation experiences	Community	aOR = 4.13; 95% CI [1.55, 10.99,] *p* = .005
Gupta et al. (2009)	Caribbean 43.1%, Africa 40.2%, South America, Central America, and Mexico 7.2%, Other 9.6%	USA	Fatherhood	Relationship	Physical IPV: *p* = .02
			Exposure to political violence	Community	Any IPV: aOR = 2.84; 95% CI [1.41, 5.74], *p* < .05 Physical IPV: aOR = 2.69; 95% CI [1.11, 6.54], *p* < .05 Sexual IPV: aOR = 2.37; 95% CI [1.04, 5.44], *p* < .05
Gupta et al. (2010)	N/R	USA	Low English-speaking ability	Individual	aOR = 2.67, 95% CI [1.43, 4.97]
			Time in new country	Individual	aOR = .47, 95% CI [0.24, 0.91]
Jin et al. (2007)	China	USA	Marital dissatisfaction	Relationship	*r* = .32, *p* < .05
			CFV: emotional abuse	Relationship	*r* = .45, *p* < .01
			CFV: physical abuse	Relationship	*r* = .56, *p* < .01
			CFV: sexual abuse	Relationship	*r* = .45, *p* < .01
			CFV: witnessing parents’ marital violence	Relationship	*r* = .31, *p* < .05
			PA: wife beating is justified	Individual	*r* = .42, *p* < .01
			PA: wives gain from beating	Individual	*r* = .26, *p* < .05
			PA: help should not be given	Individual	*r* = .26, *p* < .05
			PA: the offender is not responsible	Individual	*r* = .26, *p* < .05
Jin et al. (2008)	China	USA	Hostile attributional bias	Individual	*t*(115) = –3.52, *p* = .001
			Spouse’s aggressive language	Individual	*t*(102) = 2.40, *p* = .018
Jin and Keat (2010)	China	USA	Income change post-migration	Individual	*t*(110) = −4.45, *p* < .01
			Education change post-migration	Individual	*t*(112) = −4.01, *p* < .01
J. Y. Kim and Sung (2000)	Korea	USA	Male dominant marital power	Relationship	β = 1.292, *SE* = .44, *p* = .003
			Stress	Individual	Medium stress: β = 2.0511, *SE* = .76, *p* = .0072 High stress: β = 3.0852, *SE* = .76, *p* = .0001
I. J. Kim and Zane (2004)	Korea	USA	Anger expression	Individual	*r* = .40, *p* < .01
			Anxious adult attachment style	Individual	*r* = .42, *p* < .01
Maldonado et al. (2020)	Latin America	USA	Education	Individual	*r* = −.05, *p* < .05
			Post-traumatic stress symptoms	Individual	*r* = .18, *p* < .001
			Alcohol dependence	Individual	*r* = .24, *p* < .001
			Drug dependence	Individual	*r* = .08, *p* < .05
			Acculturation through language	Individual	*r* = .06, *p* < .05
Nam et al. (2020)	North Korea	South Korea	Stress	Individual	β = .233, *SE* = .068, *p* < .01
			Tolerant attitudes toward violence	Individual	β = .813, *SE* = .334, *p* < .05
Rothman et al. (2007)	N/R	USA	Income	Individual	*X*^2^ = 33.78, *p* < .001
			Lower education levels	Individual	*X*^2^ = 33.61, *p* < .001
			Employment	Individual	*X*^2^ = 43.71, *p* < .001
			Relationship status	Relationship	*X*^2^ = 13.40, *p* < .01
			Fatherhood	Relationship	*X*^2^ = 8.42, *p* < .01
			Criminal record	Individual	*X*^2^ = 281.61, *p* < .001
			Alcohol abuse	Individual	*X*^2^ = 64.22, *p* < .001
			Drug abuse	Individual	*X*^2^ = 91.31, *p* < .001

*Note*. N/A = not applicable; N/R = not reported; CFV = childhood family violence; PA = positive attitudes toward IPV; OR = odds ratio; aOR = adjusted odds ratio; IPV = intimate partner violence.

**Table 3.** Prominent Themes of Included Qualitative Studies of Factors Affecting IPV Perpetration Among Migrant Men.

Authors (Year)	Country of Origin	Country Post-Migration	Prominent Themes	Level
Grzywacz et al. (2009)	Mexico	USA	Jealousy	Relationship
			Traditional gender roles	Societal
			Norms of couple behavior	Societal
Montalvo-Liendo et al. (2018)	Mexico	USA	Childhood family violence	Relationship
			Infidelity	Relationship
			Norms of violence	Societal
Saez-Betacourt et al. (2008)	Mexico 87% Guatemala 13%	USA	Work-related stress	Individual
			Anger and lack of self-control	Individual
			Alcohol and drug addiction	Individual
			Jealousy	Relationship
			Infidelity	Relationship
			Legal policies and practices	Societal
			Traditional gender roles	Societal
Welland and Ribner (2010)	Mexico	USA	Norms of violence	Societal
			Traditional gender roles	Societal

*Note*. IPV = intimate partner violence.

#### Individual Level

IPV perpetration was associated with migrant men’s socioeconomic status (SES), including factors of education, employment, income, food insecurity, and living conditions. Three studies showed that lower levels of education and lack of further education post-migration were associated with higher rates of IPV perpetration (Jin & Keat, 2010; Maldonado et al., 2020; Rothman et al., 2007), whereas two studies did not find a significant relationship between education and perpetration (Gupta et al., 2009; Nam et al., 2020). Chinese American perpetrators were significantly less likely to report income increases post-migration compared to their non-violent counterparts (Jin & Keat, 2010), while migrants in a perpetrator intervention program were 25% more likely to be employed and earned higher incomes than non-migrants (Rothman et al., 2007). In contrast, three studies found no significant relationship between income and perpetration (Gilbert et al., 2019; Gupta et al., 2009; Nam et al., 2020). Immigrant men who suffered from food insecurity and lived in poor conditions were more likely to perpetrate IPV (Gilbert et al., 2019).

Demographic factors of age, time in new country, English-speaking ability, and criminal record were also examined. Age was not a risk factor for IPV perpetration among migrant men (Gupta et al., 2009; Nam et al., 2020; Rothman et al., 2007). The impacts of time in the new country and English-speaking ability were mixed. One study showed that immigrants who had lived in the United States for 6 years or more were significantly more likely to perpetrate IPV than those who had lived in the United States for 5 years or less (Gupta et al., 2010), whereas another study did not find any relationship between length of time in the United States and IPV perpetration (Gupta et al., 2009). Regarding language ability of migrants in the United States, Gupta et al. (2010) found that immigrant men with low English-speaking ability were more than twice as likely to perpetrate IPV, Maldonado et al. (2020) showed that Latino immigrants who predominantly spoke English were more likely to perpetrate IPV compared to those who predominantly spoke Spanish, and Gupta et al. (2009) observed no effect of language ability. In the United States, immigrants were half as likely as non-migrants to have a criminal record prior to conviction for IPV perpetration (Rothman et al., 2007).

Studies examined the association between IPV perpetration and factors related to mental health such as post-traumatic stress symptoms (PTSS), anxiety, depression, attachment style, anger, stress, alcohol use, and drug use. Perpetration was correlated with PTSS among Latino immigrants, but not symptoms of anxiety or depression (Maldonado et al., 2020). Anxious adult attachment style was associated with perpetration among Korean American immigrants, but not avoidant adult attachment style (I. J. Kim & Zane, 2004). Anger was one of the most cited factors for precipitating IPV for Latino perpetrators (Saez-Betacourt et al., 2008), and anger expression was related to perpetration among Korean American migrants, but not anger experience (I. J. Kim & Zane, 2004). Evidence for the impact of self-control in the context of anger was mixed; lack of self-control was frequently cited as a contributing factor by Latino IPV perpetrators (Saez-Betacourt et al., 2008), but there was no significant relationship between IPV and anger control among Korean American migrants (I. J. Kim & Zane, 2004).

Korean American immigrants and North Korean refugees with higher stress levels were more likely to perpetrate IPV (J. Y. Kim & Sung, 2000; Nam et al., 2020). Among Korean American immigrants, 38% of high stress couples experienced physical IPV in the past year, compared to only 2% of low stress couples (J. Y. Kim & Sung, 2000). A Latino migrant associated his IPV perpetration with work-related stress: “I worked from 5 a.m. to 5 p.m., Saturdays included, and my wife worked two jobs. We almost didn’t see each other during the week, we really didn’t share time together” (Saez-Betacourt et al., 2008, p. 139). Symptoms of alcohol dependence and drug dependence placed Latino immigrants at higher risk of perpetration (Maldonado et al., 2020); however, migrant men in general were half as likely as non-migrant men to report a history of alcohol abuse (18% vs. 36%), and almost five times less likely to report a history of drug abuse (Rothman et al., 2007). Several Latino perpetrators attributed their abusive behaviors to alcohol and drug addiction; one participant stated: “About 11 p.m. I was coming home drunk and I saw my wife sleeping. I woke her up and asked her to make a meal for me; when she refused, I hit her” (Saez-Betacourt et al., 2008, p. 139).

Results showed associations between IPV perpetration and men’s attitudes and cognitive bias. Positive attitudes toward IPV were associated with IPV perpetration among Chinese immigrants and North Korean refugees (Jin et al., 2007; Nam et al., 2020). Specific beliefs associated with IPV perpetration were as follows: IPV is justified, wives benefit from IPV, help should not be given to the victim, and the perpetrator is not responsible for IPV (Jin et al., 2007). Jin et al. (2008) analyzed differences between Chinese immigrant perpetrators and non-perpetrators in hostile attributional bias (HAB), the tendency to interpret ambiguous actions performed by others as having a hostile intent. Perpetrators displayed significantly less HAB than their non-perpetrating counterparts; however, a more covert measure of HAB showed that perpetrators rated their partners’ language as significantly more aggressive (Jin et al., 2008).

#### Relationship Level

Relationship factors considered all proximal social relationships, including relationships with intimate partners, family members, and the family of origin. Factors specific to the intimate relationship that were explored in the included studies were relationship status, marital satisfaction, power dynamics, jealously, and infidelity. Migrant perpetrators were more likely to be married or in a relationship than non-migrant perpetrators (Rothman et al., 2007); however, marital status did not predict past-year IPV perpetration among migrants (Gupta et al., 2009). Marital dissatisfaction was significantly associated with increased risk of IPV perpetration among Chinese migrant perpetrators (Jin et al., 2007). Among Korean immigrants, male dominant couples (i.e., men having the final say) had an increased likelihood of husband IPV perpetration compared to egalitarian couples (King & Sung, 2000). Chinese immigrant perpetrators reported having less decision-making power than their spouses after migration; however, there was no significant difference in power loss between perpetrators and non-perpetrators (Jin & Keat, 2010).

Migrant men’s jealousy was explored as a risk factor for IPV perpetration. In Israel, Ethiopian migrants (87%) were significantly more likely to commit IPH due to jealousy motives compared to Israeli natives (77%) and Russian migrants (66%; Edelstein, 2018). Jealousy motives related to the perpetrator’s sexual possessiveness and the partner’s willingness to leave the relationship (Edelstein, 2018). Mexican perpetrators experienced jealousy when they observed their partners interacting with male colleagues (Grzywacz et al., 2009), and a Latino immigrant experienced jealousy due to their partner’s infidelity (Saez-Betacourt et al., 2008). Infidelity was identified as a factor for IPV perpetration in two qualitative studies (Montalvo-Liendo et al., 2018; Saez-Betacourt et al., 2008). A Latino immigrant discussed his response to his partner’s infidelity: “I confronted her and she denied her fault. I lost control and started yelling at her, then I threw the telephone on the wall and I broke it, after breaking the wall she called the police and they arrested me” (Saez-Betacourt et al., 2008, p. 139). A Mexican immigrant stated that his own infidelity caused a confrontation with his wife; his partner told him, “If I wanted to be with her I needed to step up to the plate and be more of a man and that was the first time I was physical with her” (Montalvo-Liendo et al., 2018, p. 461).

Three studies explored the association between IPV perpetration and fatherhood. Immigrant men with children with more likely to perpetrate IPV than immigrant men without children (Gupta et al., 2009), but migrant perpetrators were still less likely to be fathers compared to non-migrant perpetrators (Rothman et al., 2007). Among Latino men, perpetration was associated with low parenting competence but not parenting stress (Baker et al., 2001).

Experiencing CFV in the family of origin, including victimization of child abuse and witnessing violence between parents, was an influential factor on adult IPV perpetration among migrant men. In Spain, immigrant perpetrators (56.6%) were significantly more likely to report CFV than native perpetrators (43.4%; Fernández-Montalvo et al., 2022). Among Chinese American perpetrators, IPV perpetration was significantly correlated with witnessing parental marital violence as a child and experiencing childhood physical, emotional, and sexual abuse (Jin et al., 2007). The most prevalent experiences of CFV among Chinese American perpetrators were witnessing violence (75.8%), childhood emotional abuse (59.0%), and childhood physical abuse (44.3%; Jin et al., 2007). A Mexican immigrant described the impact of abuse from his mother in childhood and adolescence on his behavior: “The same disrespect I had with my mother triggered the same way in my marriage” (Montalvo-Liendo et al., 2018, p. 461). On the contrary, no relationship was found between IPV perpetration and CFV among North Korean refugees (Nam et al., 2020).

#### Community Level

Community level factors that were explored among migrant perpetrators included exposure to political and social violence, deportation experiences, arrest by migration police, and racial discrimination. Immigrant market workers in Kazakhstan who had been arrested, incarcerated, or beaten for political activities or beliefs had significantly increased odds of IPV perpetration (Gilbert et al., 2019). Similarly, immigrants in the United States who had directly experienced or witnessed political violence committed by the police, army, or other political groups in their birth country, were more likely to have perpetrated past-year physical and sexual IPV (Gupta et al., 2009). In contrast, North Korean refugees who had witnessed or experienced social violence at school, in the military, the workplace, or the local community, did not have elevated risk of IPV perpetration (Nam et al., 2020). Among immigrant market workers in Kazakhstan, experience, threat, or fear of deportation significantly increased the odds of IPV perpetration; however, being arrested by migration police had no impact on perpetration (Gilbert et al., 2019). For Latino immigrants, the experience of racial/ethnic discrimination was not directly associated with perpetration (Maldonado et al., 2020).

#### Societal Level

Societal factors from the perpetrators’ countries of origin included norms of violence, traditional gender roles, and legal policies and practices. Latino perpetrators associated IPV perpetration with norms of violence in their countries of origin, which emphasized force, domination, and control as the only acceptable behaviors for men (Montalvo-Liendo et al., 2018; Welland & Ribner, 2010). A Mexican immigrant reflected, “everything was dealt with some sort of violence. There are men in my family that I would see that they were strong-minded but trying to do everything with force” (Montalvo-Liendo et al., 2018, p. 461). A specific element of Latino culture associated with IPV perpetration was *machismo*, an extreme version of the male gender role characterized by domination over women (Saez-Betacourt et al., 2008; Welland & Ribner, 2010). Latino perpetrators described *machismo* as a negative attribute, such that men were “controlling, jealous, disrespectful, incommunicative, alcoholic, feel entitled to male privilege, and be overly concerned with being respected by his wife and children” (Welland & Ribner, 2010, p. 805). Regarding the influence of *machismo* on IPV perpetration, one Latino immigrant reflected, “I believed that because I was a man I had the right to offend my partner and to do whatever I wanted to her. I was abusive, I drunk too much and I came home aggressively” (Saez-Betacourt et al., 2008, p. 139). Conversely, for North Korean refugees, belief in traditional gender roles did not predict IPV perpetration (Nam et al., 2020).

Legal policies and practices in some countries of origin were associated with IPV perpetration, specifically in Latin America where sanctions for perpetration were minimal to none (Saez-Betacourt et al., 2008). For example, a Latino immigrant reported: “violence in my country [had] a very different outcome. In my country she had tolerated the abuse. In my country the laws are not like here. Violence is a very common issue and nobody would think it was important” (Saez-Betacourt et al., 2008, p. 140). Another Latino immigrant disclosed, “in my country, the police wouldn’t arrest me. They would allow me to stay at home. As a couple we would [. . .] go to family court and the judge would ask us if we want to continue living together” (Saez-Betacourt et al., 2008, p. 140).

Adjusting to changes in traditional gender roles and norms of couple behavior in the host country were associated with IPV perpetration among migrant men. For Mexican migrant men in the United States, their partners’ employment post-migration contributed to conflict by challenging traditional gender roles surrounding the division of household labor and financial decision-making (Grzywacz et al., 2009). Mexican men reported that their partners were less available, household chores were incomplete when they returned from work, and their own attempts to engage in household activities were heavily criticized by their partners (Grzywacz et al., 2009). One participant shared, “she yells at me too much. I help out a lot in my house. And that bothers me because I know that in the majority of the Mexican marriages this doesn’t happen” (Grzywacz et al., 2009, p. 1,205). Women’s financial independence contributed to conflict among Mexican couples, especially when women spent their own money without consulting their husbands: “when she got her check, we seldom sat down and discussed what we were going to do with the money. And that’s when we would start to argue. As the head of the household, it makes me feel bad” (Grzywacz et al., 2009, p. 1,205). Changes in norms relating to couple behavior were a source of conflict within Mexican couples, as the men believed that their partners were abandoning cultural norms by trying to introduce new ideas (Grzywacz et al., 2009).

## Discussion

This review examined factors associated with IPV perpetration among first-generation migrant men. The 18 included studies covered a range of factors associated with IPV perpetration at the individual, relationship, community, and societal levels. The results included factors that were consistent with the extant literature on risk factors for IPV perpetration among men in the general population and expanded upon the literature by revealing unique factors for migrant men. The findings are summarized and interpreted below and integrated with the current literature and theoretical explanations.

For many migrant men, IPV perpetration was linked to the experience of significant socioeconomic disadvantage. Perpetrators generally had lower levels of education, were less likely to advance in education and income post-migration, experienced food insecurity, and had poor living conditions (Gilbert et al., 2019; Jin & Keat, 2010; Maldonado et al., 2020; Rothman et al., 2007). This was consistent with extant literature that associated male to female IPV with lower SES (Assari & Jeremiah, 2018; Bhona et al., 2019), lower levels of education (El-Moslemany et al., 2022), food insecurity (Schwab-Reese et al., 2016), and poverty (Gillum, 2019). Further evidence of socioeconomic disadvantage was the significant occupational and economic stress experienced by migrant perpetrators (J. Y. Kim & Sung, 2000; Saez-Betacourt et al., 2008), and the lack of parenting support reported by almost 70% of Latino men (Baker et al., 2001). As per Farrington’s (1986) stress theory, these findings suggest that the combination of occupational and familial demands and a lack of social resources and support may overwhelm the response capabilities of migrant men and increase their risk of IPV perpetration.

Despite the trend of migrant perpetrators having lower SES, there was heterogeneity in the results. Some migrant perpetrators had better SES outcomes compared to non-migrant perpetrators, that is, they were more likely to be employed, earned higher incomes, and were less likely to have prior criminal records (Rothman et al., 2007). While the occupations of these migrant men were unclear, other research on migrants in the workplace shows that they are more likely to be underemployed (i.e., employed at a lower level than their educational qualification permits), leading to lower overall well-being (Satyen et al., 2016). In addition, workplaces may be a source of distress for migrant men; migrant men’s experiences of lack of power and disrespect in the workplace have been shown to contribute to partner abuse (Hancock & Siu, 2009).

Expanding upon the extant literature of factors associated with male IPV perpetration in the general population (see Capaldi et al., 2012; Clare et al., 2021; Fulu et al., 2013; C. M. Spencer et al., 2022), this review revealed unique factors for migrant men’s IPV perpetration at the community and societal levels. Unique factors were exposure to political violence, deportation experiences, and legal policies and practices in some countries of origin where sanctions for perpetration were minimal to none. Associations between IPV perpetration and exposure to political violence and deportation experiences demonstrated the impact of traumatic experiences both pre- and post-migration (Gilbert et al., 2019; Gupta et al., 2009). Such associations may be explained by the mental health consequences of stressful and traumatic experiences; for example, Sangalang et al. (2019) showed that pre-migration trauma among migrants and refugees increased the risk of depressive disorders, anxiety disorders, and psychological distress. Legal policies and practices discussed by Latino migrants indicated that IPV perpetration was not harshly punished in their countries of origin and women were expected to tolerate the abuse (Saez-Betacourt et al., 2008). This finding suggests that legal practices in the countries of origin perpetuated the cycle of abuse, and that intervention only occurred once the men migrated and perpetrated IPV in the United States. Such patterns have also been observed in Australia, where migrant men believed that IPV was not fairly assessed and that Australian laws favored women over women (Satyen et al., 2020).

Other factors impacting on mental health that were associated with IPV perpetration among migrant men were exposure to CFV (Fernández-Montalvo et al., 2022; Jin et al., 2007), trauma symptoms (Maldonado et al., 2020), anger (I. J. Kim & Zane, 2004; Saez-Betacourt et al., 2008), alcohol and drug use (Maldonado et al., 2020; Saez-Betacourt et al., 2008), and anxious adult attachment style (I. J. Kim & Zane, 2004). Such factors were previously associated with male IPV perpetration in systematic reviews and meta-analyses (Clare et al., 2021; Kimber et al., 2018; S. Li et al., 2020; C. Spencer et al., 2019; C. M. Spencer et al., 2022). Impacts of mental health factors for migrant men are often exacerbated by low rates of service access. Barriers to help-seeking include stigma toward mental illness in some cultures, mistrust of Western mental health systems, difficulty accessing support services, and a preference to cope on their own (Amri & Bemak, 2013; Carey et al., 2022; Fortuna et al., 2008; Selkirk et al., 2014).

Several factors at the individual, relationship, and societal levels provided evidence for the impact of patriarchal values on IPV perpetration among migrant men. IPV perpetration in the included studies was associated with positive attitudes toward IPV (Jin et al., 2007; Nam et al., 2020), male dominance in the intimate relationship (Kin & Sung, 2000), and traditional gender roles (Saez-Betacourt et al., 2008; Welland & Ribner, 2010). Prior research shows that such ideologies and practices are commonly found in patriarchal cultures (Lin et al., 2016; Yoshihama et al., 2014; Zark & Satyen, 2021) and are significant predictors of IPV perpetration (Khawaja et al., 2008; Ozaki & Otis, 2017). Relationship problems and post-migration changes in the intimate relationship were also associated with IPV perpetration in the included studies, including marital dissatisfaction, jealousy, infidelity, changes to traditional gender roles, their partners’ employment and financial independence, and the beliefs that their partners had abandoned norms of couple behavior and were using aggressive language (Edelstein, 2018; Grzywacz et al., 2009; Jin et al., 2007, 2008; Montalvo-Liendo et al., 2018; Saez-Betacourt et al., 2008). Taken together, these factors indicate that migrant men can experience a loss in power and control in their intimate relationships post-migration. Such changes may threaten the patriarchal values of their cultures of origin and IPV may be perpetrated to regain power in the relationship. Indeed, research shows that among couples who experienced an escalation in conflict post-migration, men perceived a reduction in power and status, whereas women’s rights and positions improved (Darvishpour, 2002).

At the societal level, Latino men associated their IPV perpetration with traditional gender roles and norms of violence in their countries of origin (Montalvo-Liendo et al., 2018; Saez-Betacourt et al., 2008; Welland & Ribner, 2010). These factors appear to be interwoven, as the male gender role for Latino men, *machismo*, promoted violent behavior for men and contributed to community environments saturated with violence. The traditional gender roles of *machismo* and *marianismo* in Latino culture are risk factors for IPV, as the negative aspects of *machismo*, including male aggression and dominance, are reinforced by *marianismo*, which dictates that Latina women should be self-sacrificing and devote their lives to their family (Galanti, 2003; Mancera et al., 2017; Torres et al., 2002). Research shows that Latinas who adhere to traditional *marianismo* ideals are more accepting of IPV and the perpetrator, and less approving of seeking help (Dietrich & Schuett, 2013). Regarding cultural norms, Farrington (1986) argued that IPV was condoned by social norms that legitimize violence. Such norms reinforce that IPV is an appropriate and effective means of achieving a goal, and an acceptable response to frustration (Farrington, 1986).

### Strengths and Limitations

This review has both strengths and limitations. The strengths include the unique study objective of focusing on IPV perpetration among migrant men, the development of a comprehensive systematic search strategy to identify relevant studies, and systematic assessment of methodological quality using three study design specific tools. All included studies were assessed to be of good or fair quality. Quantitative and qualitative research were included in the review, allowing for the inclusion of generalizable data and rich insights from migrant men. All data were self-reported by migrant men, allowing for focus on male perspectives.

There were, however, several limitations of this review. Almost all the studies (94%) were cross-sectional, which prevents understanding of directionality, that is, determining whether factors were present prior to IPV perpetration or were an outcome of IPV. Most studies (61%) were conducted in the United States with Asian or Latino immigrants exclusively; therefore, results for each factor should not be generalized to all migrant men, and instead considered in the cultural contexts of the relevant samples. Only one study (6%) was conducted with refugees, which prevented this review from discussing differences in IPV factors between refugees and migrants. Most factors identified in the review were explored by relatively few studies and only the significant factors have been discussed. All studies assessed physical violence using the CTS, but there was heterogeneity in the measures of psychological and sexual violence. None of the included studies used IPV measures for controlling behaviors and economic/financial abuse, which may have their own unique risk factors. The studies only included men in heterosexual relationships, which limits understanding of IPV perpetration among migrant men in queer or same-sex relationships. Studies published in languages other than English were also excluded, which may have systematically excluded literature from non-English journals; this is an important limitation given the emphasis on culture in the research.

### Implications for Practice, Policy, and Research

The implications for practice, policy, and research are summarized in Table 4. Prevention and intervention programs for IPV among migrant men should be culturally sensitive and relevant and promote gender-equitable attitudes and behaviors where possible. Screening tools for IPV among migrant men should evaluate demographic and socioeconomic factors, including education, income, fatherhood, time in the new country, and language ability. Screening tools should also include assessment of trauma, such as exposure to political violence, deportation experiences, and CFV.

**Table 4.** Summary of Implications for Practice, Policy, and Research.

Type	Implications
Practice	Prevention and intervention programs for IPV among migrant men should be culturally sensitive and relevant and promote gender-equitable attitudes and behaviors where possible. Screening tools should assess education, income, fatherhood, time in the new country, and language ability. Exposure to trauma should be assessed, including political violence, deportation experiences, and CFV. Individual-level modifiable factors that should be addressed include positive attitudes toward IPV, trauma symptoms, stress, anger, alcohol use, drug use, and attachment style. Modifiable factors relating to the intimate relationship include relationship dissatisfaction, power dynamics, jealousy, infidelity, and post-migration changes to traditional gender roles and norms of couple behavior. A modifiable factor relating to family relationships was migrant men’s low parenting competence. Societal factors that were particularly relevant for Latino migrant men were traditional gender roles such as *machismo* and norms of violence.
Policy	Policymakers should fund culturally appropriate programs aimed at preventing and reducing IPV perpetration among migrant men.
Research	Culturally appropriate measures of IPV perpetration for different immigrant groups should be created and validated. Factors should be explored within specific cultures rather than across broad cultural groupings to determine culture-specific factors. Clarification is needed whether there are unique factors for IPV perpetration among different migrant status groups. Factors specific to migrant men in the LGBTIQA+ community and in same-sex relationships should be explored.

*Note*. CFV = childhood family violence; IPV = intimate partner violence.

Service providers and practitioners who deliver perpetrator intervention programs for migrant men should address individual-level modifiable factors such as positive attitudes toward IPV, trauma symptoms, stress, anger, alcohol use, drug use, attachment style, and parenting competence. Where appropriate and safe for their partners, modifiable factors that should be addressed in relationship counselling include relationship dissatisfaction, power dynamics, jealousy, infidelity, and post-migration changes to traditional gender roles and norms of couple behavior. A modifiable factor relating to family relationships was migrant men’s low parenting competence. Evidence from the included studies suggested that prominent societal factors for IPV perpetration among Latino migrant men were traditional gender roles such as *machismo* and norms of violence in the countries of origin. Policymakers should fund culturally appropriate programs aimed at preventing and reducing IPV perpetration among migrant men.

This review provides preliminary insights into factors associated with IPV among migrant men. Reliability of IPV research among migrants could be enhanced through the creation and validation of standardized, culturally appropriate measures of IPV perpetration for different immigrant groups. Future research exploring IPV among migrants should be conducted within specific cultures rather than across broad cultural groupings to determine culture-specific factors. Further research is needed to clarify whether there are unique factors associated with IPV perpetration for men from different migrant statuses, for example, migrants, refugees, and asylum seekers. The current body of research should be expanded by exploring factors associated with IPV that are specific to migrant men in the LGBTIQA+ community and in same-sex relationships.

## Conclusion

This is the first systematic review of factors associated with IPV perpetration among migrant men. The review found that a wide range of factors were associated with IPV perpetration at the individual, relationship, community, and societal levels. Specifically, the review revealed unique factors for migrant men’s IPV perpetration, including exposure to political violence, deportation experiences, and minimal legal sanctions for perpetration in some countries of origin. Societal factors explored among Latino migrant men were traditional gender roles such as *machismo* and norms of violence. The findings highlight that prevention and intervention strategies for IPV perpetration should be modified for the unique circumstances of migrant men and should target culture-specific factors. Future research should explore factors that impact on migrant men’s IPV perpetration by focusing within specific cultures rather than across broad cultural groupings.

## Supplemental Material

sj-docx-1-tva-10.1177_15248380231178758 – Supplemental material for Factors Associated with Intimate Partner Violence Perpetration Among Migrant Men: A Systematic Review Supplemental material, sj-docx-1-tva-10.1177_15248380231178758 for Factors Associated with Intimate Partner Violence Perpetration Among Migrant Men: A Systematic Review by Matin Ayubi and Lata Satyen in Trauma, Violence, & Abuse
