# Supplementary material for: Factors Associated with Intimate Partner Violence Perpetration Among Migrant Men: A Systematic Review
Source: Trauma Violence Abuse. 2023 Jun 10;25(2):1365–81. doi: 10.1177/15248380231178758 (PMC10913363; doi:10.1177/15248380231178758)
Supplement: sj-docx-1-tva-10.1177_15248380231178758 – Supplemental material for Factors Associated with Intimate Partner Violence Perpetration Among Migrant Men: A Systematic Review [file sj-docx-1-tva-10.1177_15248380231178758.docx]

**Appendix A**

**Search Terms for Titles and Abstracts**

| Concept | Search terms |
| --- | --- |
| Men | male*; man; men; husband*; boyfriend* |
| Migrants | “asylum seek*”; CALD; cultur*; “cultur* and linguistic* divers*”; divers*; ethnic*; immigra*; migra*; minorit*; race; racial; refugee*; foreign* |
| Intimate partner violence | (“intimate partner”; marital; marriage; partner; domestic; couple; dating; emotional; physical; psychological; financial; relationship; sexual; spous*; gender*; “gender based”; “inter partner”; interpartner; family; wife; wom?n) N2 (abus*; violen*; assault; conflict; aggression; batter*); “control* behav*”; “coercive control*”; DV; IPV |
| Perpetration | abusive; abuser*; aggressor*; aggression; batterer*; offender*; perpetrat* |

Note. N2 finds the terms if they are within two words of one another regardless of the order in which they appear.

**Appendix B**

**Subject Headings and Limiters for Database Searches**

| Database | Subject headings | Limiters |
| --- | --- | --- |
| MEDLINE Complete | “Men+”; Spouses; Refugees; “Transients and Migrants”; “Emigrants and Immigrants+”; Criminals; “Criminal Behavior+”; “Intimate Partner Violence+”; “Sex Offenses+”; “Gender-Based Violence”; “Domestic Violence+”; Rape; “Physical Abuse” | English Language; Scholarly (Peer Reviewed) Journals |
| Embase | Migrant; Offender; “Antisocial Behavior”; “Dating Violence”; “Domestic Violence”; “Gender Based Violence” | English Language; Source: Embase |
| PsycInfo | “Human Males”; “Human Migration”; Refugees; Immigration; “Minority Groups”; Perpetrators; “Criminal Offenders”; “Antisocial Behavior”; “Criminal Behavior”; “Intimate Partner Violence”; “Domestic Violence”; “Battered Females”; “Sexual Violence”; “Violent Crime”; “Physical Abuse”; “Dating Violence”; “Sex Offenses”; “Sexual Harassment” | English Language; Peer Reviewed |
| SocINDEX with Full Text | Men; Refugees; Immigrants; Minorities; “Emigration & immigration”; “Intimate Partner Violence”; “Dating Violence”; “Marital Violence”; “Abusive Relationships”; “Family Violence”; “LGBT Family Violence”; “Spouses -- Wounds & Injuries”; “Victims of Family Violence”; “Family Conflict”; “Battering (Abuse)”; “Abusive Men”; “Male Offenders” | English Language |

**Appendix C**

**MEDLINE Complete Search Syntax**

1. TI (male* OR man OR men OR husband* OR boyfriend*) OR AB (male* OR man OR men OR husband* OR boyfriend*)

2. (MH "Men+") OR (MH "Spouses")

3. TI (“asylum seek*” OR CALD OR cultur* OR "cultur* and linguistic* divers*" OR divers* OR ethnic* OR immigra* OR migra* OR minorit* OR race OR racial OR refugee* OR foreign*) OR AB (“asylum seek*” OR CALD OR cultur* OR "cultur* and linguistic* divers*" OR divers* OR ethnic* OR immigra* OR migra* OR minorit* OR race OR racial OR refugee* OR foreign*)

4. (MH "Refugees") OR (MH "Transients and Migrants") OR (MH "Emigrants and Immigrants+")

5. TI (abusive OR abuser* OR aggressor* OR aggression OR batterer* OR offender* OR perpetrat*) OR AB (abusive OR abuser* OR aggressor* OR aggression OR batterer* OR offender* OR perpetrat*)

6. (MH "Criminals") OR (MH "Criminal Behavior+")

7. TI ((“intimate partner” OR marital OR marriage OR partner OR domestic OR couple OR dating OR emotional OR physical OR psychological OR financial OR relationship OR sexual OR spous* OR gender* OR “gender based” OR “inter partner” OR interpartner OR family OR wife OR wom?n) N2 (Abus* OR violen* OR assault OR conflict OR aggression OR batter*))

8. TI “control* behav*” OR TI “coercive control*” OR TI dv OR TI ipv

9. AB ((“intimate partner” OR marital OR marriage OR partner OR domestic OR couple OR dating OR emotional OR physical OR psychological OR financial OR relationship OR sexual OR spous* OR gender* OR “gender based” OR “inter partner” OR interpartner OR family OR wife OR wom?n) N2 (Abus* OR violen* OR assault OR conflict OR aggression OR batter*))

10. AB “control* behav*” OR AB “coercive control*” OR AB dv OR AB ipv

11. (MH "Intimate Partner Violence+") OR (MH "Sex Offenses+") OR (MH "Gender-Based Violence") OR (MH "Domestic Violence+") OR (MH "Rape") OR (MH "Physical Abuse")

12. S1 OR S2

13. S3 OR S4

14. S5 OR S6

15. S7 OR S8 OR S9 OR S10 OR S11

16. S12 AND S13 AND S14 AND S15

17. Limit S16 to English Language and Scholarly (Peer Reviewed) Journals

**Appendix D**

**Embase Search Syntax**

1. male*:ab,ti OR man:ab,ti OR men:ab,ti OR husband*:ab,ti OR boyfriend*:ab,ti

2. 'asylum seek*':ab,ti OR cald:ab,ti OR cultur*:ab,ti OR 'cultur* and linguistic* divers*':ab,ti OR divers*:ab,ti OR ethnic*:ab,ti OR immigra*:ab,ti OR migra*:ab,ti OR minorit*:ab,ti OR race:ab,ti OR racial:ab,ti OR refugee*:ab,ti OR foreign*:ab,ti

3. 'migrant'/exp

4. abusive:ab,ti OR abuser*:ab,ti OR aggressor*:ab,ti OR aggression:ab,ti OR batterer*:ab,ti OR offender*:ab,ti OR perpetrat*:ab,ti

5. 'offender'/de OR 'antisocial behavior'/exp

6. (('intimate partner' OR marital OR marriage OR partner OR domestic OR couple OR dating OR emotional OR physical OR psychological OR financial OR relationship OR sexual OR spous* OR gender* OR 'gender based' OR 'inter partner' OR interpartner OR family OR wife OR wom?n) NEAR/2 (abus* OR violen* OR assault OR conflict OR aggression OR batter*)):ab,ti

7. 'control* behav*':ab,ti OR 'coercive control*':ab,ti OR dv:ab,ti OR ipv:ab,ti

8. 'dating violence'/de OR 'domestic violence'/exp OR 'gender based violence'/de

10. S2 OR S3

11. S4 OR S5

12. S6 OR S7 OR S8

13. S1 AND S10 AND S11 AND S12

14. Limit S13 to English Language and Embase Source

**Appendix E**

**PsycInfo Search Syntax**

1. TI (male* OR man OR men OR husband* OR boyfriend*) OR AB (male* OR man OR men OR husband* OR boyfriend*)

2. DE "Human Males"

3. TI (“asylum seek*” OR CALD OR cultur* OR "cultur* and linguistic* divers*" OR divers* OR ethnic* OR immigra* OR migra* OR minorit* OR race OR racial OR refugee* OR foreign*) OR AB (“asylum seek*” OR CALD OR cultur* OR "cultur* and linguistic* divers*" OR divers* OR ethnic* OR immigra* OR migra* OR minorit* OR race OR racial OR refugee* OR foreign*)

4. DE "Human Migration" OR DE "Refugees" OR DE "Immigration" OR DE "Minority Groups"

5. TI (abusive OR abuser* OR aggressor* OR aggression OR batterer* OR offender* OR perpetrat*) OR AB (abusive OR abuser* OR aggressor* OR aggression OR batterer* OR offender* OR perpetrat*)

6. DE "Perpetrators" OR DE "Criminal Offenders" OR DE "Antisocial Behavior” OR DE "Criminal Behavior"

7. TI ((“intimate partner” OR marital OR marriage OR partner OR domestic OR couple OR dating OR emotional OR physical OR psychological OR financial OR relationship OR sexual OR spous* OR gender* OR “gender based” OR “inter partner” OR interpartner OR family OR wife OR wom?n) N2 (Abus* OR violen* OR assault OR conflict OR aggression OR batter*))

8. TI “control* behav*” OR TI “coercive control*” OR TI dv OR TI ipv

9. AB ((“intimate partner” OR marital OR marriage OR partner OR domestic OR couple OR dating OR emotional OR physical OR psychological OR financial OR relationship OR sexual OR spous* OR gender* OR “gender based” OR “inter partner” OR interpartner OR family OR wife OR wom?n) N2 (Abus* OR violen* OR assault OR conflict OR aggression OR batter*))

10. AB “control* behav*” OR AB “coercive control*” OR AB dv OR AB ipv

11. DE "Intimate Partner Violence" OR DE "Domestic Violence" OR DE "Battered Females" OR DE "Sexual Violence" OR DE "Violent Crime" OR DE "Physical Abuse" OR DE "Dating Violence" OR DE "Sex Offenses" OR DE "Sexual Harassment"

12. S1 OR S2

13. S3 OR S4

14. S5 OR S6

15. S7 OR S8 OR S9 OR S10 OR S11

16. S12 AND S13 AND S14 AND S15

17. Limit S16 to Peer Reviewed and English

**Appendix F**

**SocINDEX Search Syntax**

1. TI (man OR men OR husband* OR boyfriend*) OR AB (man OR men OR husband* OR boyfriend*)

2. DE "MEN"

3. TI (“asylum seek*” OR cultur* OR divers* OR ethnic* OR immigra* OR migra* OR minorit* OR race OR racial OR refugee* OR foreign*) OR AB (“asylum seek*” OR cultur* OR divers* OR ethnic* OR immigra* OR migra* OR minorit* OR race OR racial OR refugee* OR foreign*)

4. DE "REFUGEES" OR DE "IMMIGRANTS" OR DE "MINORITIES" OR DE "EMIGRATION & immigration"

5. TI ((“intimate partner” OR marital OR marriage OR partner OR domestic OR couple OR dating OR emotional OR physical OR psychological OR financial OR relationship OR sexual OR spous* OR gender* or “gender based” OR “inter partner” OR interpartner) W2 (abuse OR violence OR assault OR conflict OR aggression)) OR TI ipv OR TI dv OR TI controlling behav*

6. AB ((“intimate partner” OR marital OR marriage OR partner OR domestic OR couple OR dating OR emotional OR physical OR psychological OR financial OR relationship OR sexual OR spous* OR gender* or “gender based” OR “inter partner” OR interpartner) W2 (abuse OR violence OR assault OR conflict OR aggression)) OR AB ipv OR AB dv OR AB controlling behav*

7. DE "INTIMATE partner violence" OR DE "DATING violence" OR DE "MARITAL violence" OR DE "ABUSIVE relationships" OR DE "FAMILY violence" OR DE "LGBT family violence" OR DE "SPOUSES -- Wounds & injuries" OR DE "VICTIMS of family violence" OR DE "FAMILY conflict" OR DE "BATTERING (Abuse)"

8. abusive OR abuser* OR aggressor* OR aggression OR batterer* OR offender* OR perpetrat*

9. DE "ABUSIVE men" OR DE "MALE offenders"

10. S1 OR S2

11. S3 OR S4

12. S5 OR S6 OR S7

13. S8 OR S9

14. S10 AND S11 AND S12 AND S13

15. Limit S14 to English Language

**Appendix G**

**Characteristics of Measures of Exposure(s) and Outcome(s) Used in Included Studies**

| Author (year) | Exposure(s) | Measure(s) of exposure(s) | Outcome | | | |
| --- | --- | --- | --- | --- | --- | --- |
|  |  |  | Source of data | Measure(s) of IPV | Type of IPV (timeframe) | Rates of IPV perpetration |
| Baker et al. (2001) | Number of children, woman’s employment, parenting stress, parenting competence | Parenting Stress Scale, Parent Satisfaction Scale | Self-report | Conflict Tactics Scale (CTS; Form N), Psychological Maltreatment of Women Inventory (PMWI) | Physical, sexual, psychological | 100%^a^ |
| Edelstein (2018) | Separation, sexual jealousy, prolonged  conflict | Court decisions | Court files | Court decisions | Intimate partner homicide | 100% |
| Fernández-Montalvo et al. (2020) | Childhood family violence | General Structured Interview of Batterer Men | Court-referred and/or enrolled in treatment program | General Structured Interview of Batterer Men | N/R | 100% |
| Gilbert et al. (2019) | Poor living conditions; homeless; mobility; food insecurity; income below living wage; arrested, incarcerated, or beaten up for political activities or beliefs; deported, threatened to be deported, or feared being deported; arrested by migration police; childhood sexual abuse; harazrdous drinking; depression; social support | Single item questions, Stressful Life Events Questionnaire, Alcohol Use Disorders Identification Test, Brief Symptom Inventory, Enhancing Recovery in Coronary  Heart Disease Social Support Instrument | Self-report | Revised Conflict Tactics Scales  (CTS2) | Physical, sexual (6 months) | 5.8% |
|  |  |  |  |  | Physical, sexual (lifetime) | 9.8% |
| Grzywacz et al. (2009) | IPV interpretations and experiences, beliefs about gender roles before and after immigration, contribution of changing roles to conflict, perceptions of women’s employment | Semi-structured interview protocol | Self-report or community report | N/A | N/R | N/R |
| Gupta et al. (2009) | Age, race/ethnicity, region of origin, years in U.S., English-speaking ability, current marital status, has children, income, educational attainment, exposure to political violence | Single item questions, political violence exposure checklist | Self-report | CTS2, Sexual Experiences Survey | Physical, sexual (past year) | 17.9% |
|  |  |  |  |  | Physical (past year) | 9.5% |
|  |  |  |  |  | Sexual (past year) | 11.1% |
| Gupta et al. (2010) | Age, education, race/ethnicity, occupation, has children, marital status, country of origin, length of time in U.S., English-speaking ability | Single item questions | Self-report | CTS2, Sexual Experiences Survey | Physical, sexual (past year) | 16.6%^b^  23.9%^c^ |
| Jin et al. (2007) | Marital dissatisfaction, childhood emotional abuse, childhood physical abuse, childhood sexual abuse, witnessing parents' marital violence, beliefs about wife beating | Childhood Trauma Questionnaire, Inventory of Beliefs about Wife Beating, Index of Marital Satisfaction | Self-report | CTS (Form N) | Physical, psychological (past year) | 70.0%^d^ |
| Jin et al. (2008) | Hostile attributional bias (HAB), covert HAB | Ratings of spouse’s hostile motives after exposure to ambiguous emotional response scenarios, Spouse Language Use Survey | Self-report | CTS (Form N) | Physical, psychological (past year) | 70.0%^de^ |
| Jin and Keat (2010) | Attitudes toward IPV, decision power change, income change, educational change | Single item questions, Inventory of Beliefs About Wife Beating, family decision-making questions | Self-report | CTS (Form N) | Physical, psychological (past year) | 70.0%^de^ |
| Kim and Sung (2000) | Marital power type, stress | Decision Power Index, Shared Power Index, Stressful Life Events Scale | Self-report | CTS | Minor or severe physical (past year) | 18.0% |
|  |  |  |  |  | Severe physical, psychological (past year) | 6.3% |
| Kim and Zane (2004) | Anger experience, anger expression, anger control, anxious adult attachment, avoidant adult attachment | Single item questions, Nam-Powers Socioeconomic Status scores, Hispanic Stress Inventory, Short Michigan Alcohol Screening Test, Self-Construal Scale, State–Trait Anger Expression Inventory, Relationship Scales Questionnaire | Self-report | CTS | Physical | 100% |
| Maldonado et al. (2020) | Racial discrimination, anxiety symptoms, depression symptoms, post-traumatic stress symptoms, alcohol dependence symptoms, drug dependence symptoms, education, language-based, acculturation | Single item questions, Experiences of Discrimination Scale, questions corresponding to DSM-IV diagnostic criteria, Language Orientation subscale of the Short Acculturation Scale | Self-report | CTS (Form R) | Physical, psychological, sexual (past year) | 5% |
| Montalvo-Liendo et al. (2018) | Family history, first IPV incident, factors contributing to IPV, influences on men of Mexican origin to perpetrate IPV | Semi-structured interview protocol | Self-report | N/A | N/R | 100% |
| Nam et al. (2020) | Age, education, income, child abuse victimization, witnessing IPV between parents, witnessing social violence, victimization of social violence, stress, attitude toward violence, gender role attitude | Single item questions, Parent–Child Conflict Tactics Scale, CTS2, Attitude toward Violence Scale, Stressful Life Event Scale, Sex Role Attitude Scale | Self-report | CTS2 | Physical (past year) | 57.1% |
| Rothman et al. (2007) | Age, income, race/ethnicity, education, employment status, intimate partner relationship status, fatherhood status, prior criminal record, current sentence, history of alcohol abuse, history of drug abuse | Single item questions | Court-referred | N/A | N/R | 100% |
| Saez-Betacourt et al. (2008) | Relationship history, contributing factors for IPV incident, contribution of Latino culture to IPV perpetration, hypothetical consequences in county of origin | Semi-structured interview protocol | Court-referred | N/A | N/R | 100% |
| Welland and Ribner (2010) | Theories of etiology, risk factors, ecosystemic issues | Semi-structured interview protocol | Court-referred | N/A | N/R | 100% |

*Note*. N/A = not applicable. N/R = not reported.

^a^ Intervention group. ^b^ Recent migrants (< 6 years in the U.S.). ^c^ Non-recent migrants (≥ 6 years in the U.S.). ^d^ Among the perpetrator group, 100% had committed lifetime IPV and 70% had committed past-year IPV. ^e^ As reported in Jin et al. (2007).

**Appendix H**

**Quality Assessment of Included Cross-Sectional Studies**

| Authors (year) | Criteria | | | | | | | | | | | | | | Quality rating |
| --- | --- | --- | --- | --- | --- | --- | --- | --- | --- | --- | --- | --- | --- | --- | --- |
|  | 1 | 2 | 3 | 4 | 5 | 6 | 7 | 8 | 9 | 10 | 11 | 12 | 13 | 14 |  |
| Baker et al. (2001) | + | + | + | + | – | – | – | + | + | / | + | / | / | – | Fair |
| Fernández-Montalvo et al. (2020) | + | + | ? | + | + | – | – | – | + | / | + | / | / | – | Fair |
| Gilbert et al. (2019) | + | + | ? | + | – | – | – | – | + | / | + | / | / | + | Fair |
| Gupta et al. (2009) | + | + | + | + | – | – | – | – | + | / | + | / | / | + | Fair |
| Gupta et al. (2010) | + | + | + | + | – | – | – | / | + | / | + | / | / | + | Good |
| Jin et al. (2007) | + | + | – | + | – | – | – | + | + | / | + | / | / | – | Fair |
| Jin et al. (2008) | + | + | + | + | – | – | – | + | + | / | + | / | / | – | Fair |
| Jin and Keat (2010) | + | + | – | + | – | – | – | + | + | / | + | / | / | – | Fair |
| Kim and Sung (2000) | + | + | – | + | – | – | – | + | + | / | + | / | / | – | Fair |
| Kim and Zane (2004) | + | + | ? | + | – | – | – | + | + | / | + | / | / | – | Fair |
| Maldonado et al. (2020) | + | + | ? | + | – | – | – | + | + | / | + | / | / | – | Fair |
| Nam et al. (2020) | + | + | ? | + | – | – | – | + | + | / | + | / | / | – | Fair |
| Rothman et al. (2007) | + | + | + | + | – | – | – | – | + | / | + | / | / | – | Fair |

*Note.* Criteria: 1 = Was the research question or objective in this article clearly stated? 2 = Was the study population clearly specified and defined? 3 = Was the participation rate of eligible persons at least 50%? 4 = Were all the subjects selected or recruited from the same or similar populations (including the same time period)? Were inclusion and exclusion criteria for being in the study prespecified and applied uniformly to all participants? 5 = Was a sample size justification, power description, or variance and effect estimates provided? 6 = For the analyses in this paper, were the exposure(s) of interest measured prior to the outcome(s) being measured? 7 = Was the timeframe sufficient so that one could reasonably expect to see an association between exposure and outcome if it existed? 8 = For exposures that can vary in amount or level, did the study examine different levels of the exposure as related to the outcome (e.g., categories of exposure or exposure measured as a continuous variable)? 9 = Were the exposure measures (independent variables) clearly defined, valid, reliable, and implemented consistently across all study participants? 10 = Was the exposure(s) assessed more than once over time? 11 = Were the outcome measures (dependent variables) clearly defined, valid, reliable, and implemented consistently across all study participants? 12 = Were the outcome assessors blinded to the exposure status of participants? 13 = Was loss to follow-up after baseline 20% or less? 14 = Were key potential confounding variables measured and adjusted statistically for their impact on the relationship between exposure(s) and outcome(s)? Symbols: + = yes, – = no, ? = cannot determine or not reported, and / = not applicable.
